# Working memory load disrupts gaze-cued orienting of attention

**DOI:** 10.3389/fpsyg.2015.01258

**Published:** 2015-08-24

**Authors:** Anna K. Bobak, Stephen R. H. Langton

**Affiliations:** School of Natural Sciences, University of Stirling, Stirling, UK

**Keywords:** gaze-cued attention, working memory, top-down control, random number generation, executive load

## Abstract

A large body of work has shown that a perceived gaze shift produces a shift in a viewer’s spatial attention in the direction of the seen gaze. A controversial issue surrounds the extent to which this gaze-cued orienting effect is stimulus-driven, or is under a degree of top-down control. In two experiments we show that the gaze-cued orienting effect is disrupted by a concurrent task that has been shown to place high demands on executive resources: random number generation (RNG). In Experiment 1 participants were faster to locate targets that appeared in gaze-cued locations relative to targets that appeared in locations opposite to those indicated by the gaze shifts, while simultaneously and continuously reciting aloud the digits 1–9 in order; however, this gaze-cueing effect was eliminated when participants continuously recited the same digits in a random order. RNG was also found to interfere with gaze-cued orienting in Experiment 2 where participants performed a speeded letter identification response. Together, these data suggest that gaze-cued orienting is actually under top-down control. We argue that top-down signals sustain a goal to shift attention in response to gazes, such that orienting ordinarily occurs when they are perceived; however, the goal cannot always be maintained when concurrent, multiple, competing goals are simultaneously active in working memory.

## Introduction

In various social contexts, people tend to take notice of others’ gaze direction. The past two decades have seen a large number of studies investigating this social orienting phenomenon utilizing a modified version of Posner’s (1980) cueing paradigm (see Frischen et al., 2007, for a review). In this task, response times (RTs) to either detect, identify or localize targets appearing in gazed at locations (i.e., cued targets) are compared with responses to targets in locations that have not been gazed-at (i.e., uncued targets). In line with the view that people tend to pay attention to where others are looking, studies have consistently shown shorter RTs to cued than to uncued targets (e.g., Friesen and Kingstone, 1998; Driver et al., 1999; Langton and Bruce, 1999). The authors of the original studies demonstrating this gaze cueing effect argued for its reflexive, stimulus-driven nature, a claim supported by more recent evidence suggesting that the effect is immune to interference from a concurrent working memory (WM) load (Law et al., 2010; Hayward and Ristic, 2013). The aim of this paper is to revisit this recent evidence, and to investigate whether a more demanding concurrent WM task will disrupt gaze-cued orienting. Such a result would suggest that, rather than a stimulus-driven reflex, gaze cueing should be better understood as being under a degree of top down control.

Researchers have drawn a broad distinction between, on the one hand, exogenous, bottom-up, reflexive, or stimulus-driven attention, and on the other, endogenous, top-down, or wilful attention (e.g., Posner, 1980; Jonides, 1981). Several lines of evidence suggest that the gaze-cueing effect is more like the former than the latter. First, it emerges even when participants are explicitly asked to ignore the faces that provide the directional cues (Langton and Bruce, 1999); second, the gaze-cueing effect is observed when participants are aware that gaze cues do not reliably predict the locations of the forthcoming targets (i.e., targets are equally likely to appear in any of the possible target locations following any gaze cue), or even when targets are actually more likely to appear in uncued relative to cued locations (Driver et al., 1999; Kuhn and Kingstone, 2009); third, gaze cueing occurs even when participants know with 100 per cent certainty that targets will appear in a particular location (Galfano et al., 2012); and finally, gaze cues facilitate attention shifts even when a peripheral target is accompanied by an irrelevant sudden onset distractor in a mirror opposite location (Friesen et al., 2005).

Despite this compelling evidence for the stimulus-driven character of social orienting, some authors suggest that a top-down component is involved in the process (e.g., Vecera and Rizzo, 2004, 2006; Koval et al., 2005). For example, Vecera and Rizzo (2004, 2006) demonstrated that patient EVR who sustained large lesions to orbitofrontal cortex—a part of the brain linked to executive functioning—showed a normal, exogenous orienting of attention in response to sudden onset peripheral cues, but did not show an orienting response to centrally presented gaze cues. This was irrespective of how well the gazes predicted the likely location of the targets (50 and 75% accuracy). As a result of the neurological damage, EVR was also left with certain difficulties in goal directed behavior, such as typical daily activities, or decision making when presented with a problem (Vecera and Rizzo, 2004). The authors therefore argued that gaze-directed orienting is subjected to top-down modulation in a similar way to other behaviors that require sustained and selective attention to socially relevant cues, such as words and arrows. A recent study by Tipples (2008) reported that, indeed, individual differences in self-reported attentional control are linked to orienting cued by arrows and gazes, but not to orienting cued by peripherally presented sudden-onset stimuli.

Ostensibly, these neuropsychological data do seem to suggest that gaze-cued orienting is rather less like a stimulus-driven reflex and more akin to endogenous, wilful orienting of attention. However, as pointed out by Frischen et al. (2007), we should be cautious in over-interpreting these results for it is unclear whether EVR displayed a normal pattern of cueing prior to sustaining the brain lesion. Hietanen et al. (2006) pointed out that not all individuals display the typical pattern of reflexive orienting to gaze cues and EVR could have been one of them. Nevertheless, Vecera and Rizzo’s work certainly hints at top-down involvement in gaze-cued orienting.

If gaze cued attention is modulated by top-down processes, WM is the likely mechanism responsible for the modulation. Indeed, numerous studies have shown that WM is linked to attentional control in the antisaccade task (Kane et al., 2001) and that attention to visual distractors is influenced by the content of WM (Lavie and De Fockert, 2005; Spinks et al., 2004). Moreover, WM content was found to be congruent with what is attended to (Downing, 2000; Pratt and Hommel, 2003; Olivers et al., 2006; Soto et al., 2008; Olivers, 2009). WM is therefore a convincing candidate for a system controlling “endogenous” shifts of attention, which may include those made in response to gazes. However, across two experiments, Law et al. (2010) found no evidence for WM involvement in gaze cueing. While there was overall slowing of RTs to peripheral targets following a gaze cue when participants were engaged in a concurrent high load WM task (retain a five digit sequence during each gaze-cueing trial), rather than a low load WM task (retain a single digit in memory) or no concurrent secondary task, the gaze cueing effect remained intact across all secondary task conditions. A recent study by Hayward and Ristic (2013) yielded similar results: once again, gaze-cued orienting was found to be resilient to a concurrent WM load (retain a five digit sequence); however, the authors went a step further in demonstrating that their concurrent WM task did in fact disrupt endogenous orienting of attention, suggesting that gaze-cued orienting and endogenous orienting are independent processes.

In summary, although the work of Vecera and Rizzo (2004, 2006) has suggested that top-down factors might be involved in gaze-cued orienting of attention, the effect has remained stubborn to demands imposed by concurrent cognitive tasks (Law et al., 2010; Hayward and Ristic, 2013). The issue about whether gazecued orienting can best be described as an exogenous or an endogenous process therefore remains unresolved.

In this paper we revisit the finding that gaze-cued orienting is unaffected by a concurrent cognitive load. One of the problems with the digit load concurrent task used by both Law et al. (2010, Experiment 1) and Hayward and Ristic (2013) is that it does not necessarily place overly large demands on WM resources. For example, Baddeley and Hitch (1974, cited in Baddeley, 1990) showed that participants could maintain and rehearse out loud sequences of up to eight digits while simultaneously carrying out reasoning, learning and comprehension tasks, with only minimal interference; Law et al. (2010) and Hayward and Ristic (2013) each used just five digit sequences in their high load secondary tasks. Second, there is a growing body of research showing that WM is flexible and can prioritize between competing goals (see Ma et al., 2014, for a review). Pertinently, maintenance rehearsal, the resource-demanding aspect of the digit load task employed in the Law et al. (2010) and Hayward and Ristic (2013) studies, could have been suspended during the brief period when participants were performing the gaze-cueing task. To see that this could be so, consider the sequence of events on each trial in the relevant experiments reported by Law et al. (2010) and Hayward and Ristic (2013). Following the presentation of a fixation cross participants were shown the to-be-retained digit sequence for 1500 ms. The fixation cross then reappeared for 1000 ms prior to the presentation of the gazing face, which was displayed for up to 1000 ms, depending on the stimulus onset asynchrony (SOA) condition. This was followed by the presentation of the target, which demanded either a localisation response (Law et al., 2010), which averaged around 450 ms under digit load conditions, or a target detection response (Hayward and Ristic, 2013), which averaged around 400 ms. Finally, participants were given a WM prompt—a single digit from the retained sequence—to which they were asked to respond by entering the next digit in the five digit sequence. Participants could therefore have encoded the digit sequence upon its presentation and continued to rehearse this for up to 2500 ms before the gaze cue was presented. Rehearsal could then have been suspended for the duration of the presentation of the gaze cue, and the presentation and response to the target stimulus, which would have amounted to, at most, 1500 ms. During this time WM resources could have been available to initiate an attention shift in the direction of the gaze cue, producing the normal gaze-cueing effect on RTs. Rehearsal of the digit sequence could then be successfully resumed because, as shown by Baddeley (2002), material can be passively stored in WM (i.e., without rehearsal) for up to 2000 ms before decay renders it irretrievable. The sequence would therefore still be available in WM for subsequent rehearsal and response following the presentation of the memory prompt.

Our argument is therefore that, regardless of whether or not the digit load task places excessively high demands on participants’ executive resources, the demands are not necessarily imposed during the period when participants are shifting attention in response to the seen gazes. Clearly what is needed is a secondary task that must genuinely be carried out simultaneously and continuously with the gaze cueing procedure. Law et al. (2010) attempted one such task. In their second experiment participants carried out a sequence of gaze-cueing trials while at the same time listening to an auditory description of a matrix pattern, which they used to build up a mental image of the shape. Participants visualized a 5 × 3 grid of unfilled squares. They were then presented with a 15 word sequence consisting of the words “filled” and “unfilled,” which instructed them as to which of the squares on their imaginary should be filled-in, and which should be left blank. The resulting grid of filled and unfilled squares depicted one of the digits 1–9, which participants were then asked to report. This task clearly demands both manipulation and maintenance of visuospatial information, and would seem to require that processing be carried out simultaneously with the gaze cueing tasks. Gaze-cued orienting was nonetheless unaffected by this secondary task, leading the authors to conclude that it is a largely stimulus-driven reflex. However, it is possible that, as with the digit load task, participants could strategically suspend the processing aspect of the secondary task—the mental filling-in of the squares—until after the gaze tasks had been completed. The task could then become one of maintaining in memory a verbal sequence during the gaze-cueing trials. Alternatively, participants could allocate resources to building up the mental image between gaze-cueing trials, briefly suspend this while the gaze cues and targets were presented, and then resume the mental grid filling before the start of the following gaze-cueing trial. Both accounts are consistent with the account of flexible allocation of WM resources depending on the prioritized goal (Ma et al., 2014).

In the experiments reported in this paper we employed an executively demanding secondary task that must genuinely be completed concurrently with the gaze cueing procedure: random number generation (RNG). Generating random sequences from a well known and well defined set of items, such as the numbers one to nine, or letters of the alphabet, requires participants to generate and run a plan for the retrieval of an item from the appropriate set. They must keep track of the frequency with which they have generated each item, and compare sequences to some conception of randomness. If recent sequences are judged to be insufficiently random, a new strategy must be devised and initiated. In addition, well-learned or stereotypical sequences (e.g., 1-2-3-4, or A-B-C-D) must be inhibited. Random sequence generation therefore seems to draw on a range of executive processes, a claim supported by the work of Miyake et al. (2000) and Jahanshahi et al. (1998). For example, the latter group showed that transcranial magnetic stimulation of the left dorsolateral prefrontal cortex—an area associated with executive functioning—impaired participants’ ability to generate random sequences of numbers. Concurrent generation of random sequences has also been shown to have a negative effect on a range of tasks, including the learning of simple contingencies (Dienes et al., 1991); performing mental arithmetic (Logie et al., 1994); syllogistic reasoning (Gilhooly et al., 1993); choosing appropriate moves in chess, and remembering the positions of chess pieces (Robbins et al., 1996). Random number or interval generation, unlike reciting equal intervals, was reported to disrupt performance on the Corsi Blocks Task (Vandierendonck et al., 2004) and other tasks tapping into executive components of spatial WM (Towse and Cheshire, 2007).

The evidence that RNG taps executive processes, particularly those involved in spatial WM tasks, and the fact that it can be performed continuously, make it a good candidate for a secondary task with which to investigate the impact of WM on the gaze-cueing effect. In each of the experiments reported here, participants performed blocks of standard gaze-cueing trials with target localization (Experiment 1) and target identification (Experiment 2) responses. In easy secondary task conditions, participants repeatedly recited aloud the digits 1 to 9 in sequence at the rate of one digit per second while performing the gaze cueing trials. In the hard secondary task conditions, participants generated random numbers, again at the rate of one per second, from the same set of digits. Counting numbers aloud, in order, is a stereotyped response, which should not be demanding of executive resources. Gaze cued orienting, whether stimulus-driven or involving a volitional component, ought to be observed under these conditions. However, if attention shifts in response to seen gazes share executive processes with RNG, we would expect the effect to be reduced, or absent when participants are engaged in the hard secondary task.

## Experiment 1

### Materials and Methods

#### Participants

University of Stirling students and visitors (17 women, 7 men, with a mean age of 23.71 years, and range of 18–40 years) were recruited through the online sign-up system and online advertising. Psychology students were awarded experimental credits for their participation and the remaining volunteers participated on an entirely voluntary basis. All participants had self-reported normal or corrected-to-normal vision. All experimental procedures have been approved by the University of Stirling Research Ethics Committee and adhere to the principles of the 1964 Helsinki Declaration. Written informed consent was obtained from all participants.

#### Materials and Apparatus

***Primary gaze cueing task***

A color photograph of a male face with neutral facial expression cropped of all external features subtending 5.7 × 3.7° of visual angle was used in the experiment. The face model was selected from the Radboud Faces Database (Langner et al., 2010), and the stimuli were prepared using Adobe Photoshop 7.0. A cross was used as a fixation point at the beginning of each trial, subtending 0.3°. The stimulus employed as the target was a white asterisk subtending 0.3° and located at the same level as the eyes 5 cm (4.1°) from the midpoint of the photograph to the left or right.

***Secondary task***

In the secondary tasks participants were required to produce random sequences of numbers from 1 to 9 in the hard condition, or, in the easy condition, recite out loud the digits from 1 to 9 in sequence at the rate of one digit per second. The pace was indicated by a JOYO JM-65 metronome. Sequences were recorded using Olympus VN-5500 Digital Voice Recorder to ensure that participants were, indeed, performing the relevant secondary task.

All stimuli were presented against a black background on a 17-inch monitor set to 1152 × 864 pixels and refreshing at the rate of 75 MHz using E-Prime software (Psychology Software Tools, Pittsburgh, PA, USA). Reaction times and responses to targets were registered using a Serial Response Box (Psychology Software Tools, Pittsburgh, PA, USA).

#### Design

The experiment employed a within-subjects design with three independent variables: cue validity (cued, uncued), secondary task (hard, easy), and (SOA, 300 ms, 1000 ms). The dependent variable was RT in response to targets.

#### Procedure

All participants were seated 70 cm away from the computer screen in a dimly lit room. Participants performed the secondary tasks concurrently with the gaze trials. In the hard secondary task condition, participants were asked to imagine an infinite number of numbers from one to nine in a hat and pulling them out one at a time, replacing each after it has been read. They were asked to generate the numbers out loud at a rate of one per second indicated by the sound of a metronome and informed that their voice was to be recorded for the purpose of further analysis. In the easy secondary task participants were instructed to recite the digit sequence from 1 to 9 repeatedly at a rate of one digit per second. Again, participants were asked to keep pace with the metronome, and informed about the active recording of their voice.

**FIGURE 1:**
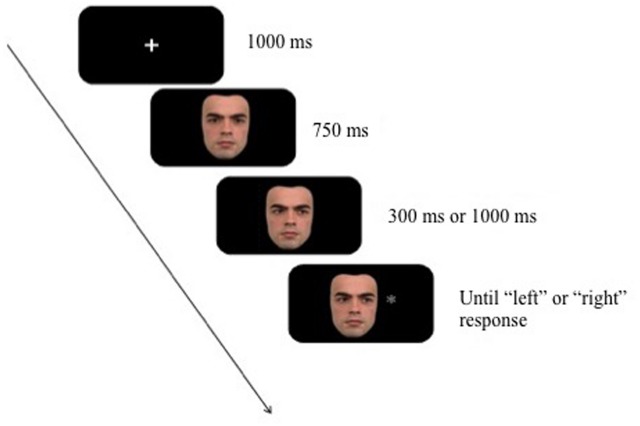
**Example trial sequence from Experiment 1 (not drawn to sale)**.

An example of a gaze cueing trial is illustrated in Figure 1. All trials began with a fixation cross displayed on the screen for 1000 ms. This was followed by a directly gazing face for 750 ms after which the gaze shifted to the left or right. The gaze cue was displayed for either 300 ms or 1000 ms before the onset of the target stimulus (i.e., the SOA). The gaze cue was non-predictive of the location (i.e., 50% cued and 50% uncued trials). Both the cue and the target remained on screen until response. Participants were asked to press the right foremost button on the serial box for targets appearing on the right side of the face and the left foremost button for targets appearing on the left.

Participants completed a set of four blocks of 32 trials under each of the secondary task conditions. These comprised 16 repetitions of the factorial combinations of cue validity (cued, uncued), SOA (300 ms, 1000 ms), and gaze direction (left, right). Whether participants began with a set of four blocks of trials under easy or hard secondary task conditions was counterbalanced between participants. Prior to starting each set of four blocks, participants completed a block of 16 practice trials. Blocks in each set of four consisted of trials drawn randomly, without replacement from the pool of 128 trials. Participants were given five seconds before the first trial in each block to begin reciting the appropriate digit sequence (i.e., random or sequential).

Volunteers were informed that the gaze direction of the displayed face did not reliably predict the future localization of the target stimulus and advised that both tasks were of equal importance and that they should aim to maximize performance on each of the tasks.

### Results

Gaze cueing trials with errors were removed from analysis, resulting in the loss of 1.47% of the data. From the remaining data, median RTs were computed for each participant in each condition of the experiment. The interparticipant means of these RTs are recorded in the top row of Table 1. The data clearly violated the homogeneity of variance assumption (Hartley’s *F*_max_ = 8.77, *p* < 0.01). A transformation of the data was therefore performed by computing the reciprocal of each participant’s median RT in each condition of the experiment. This transformation was found to stabilize the variances (Hartley’s *F*_max_ = 2.10, *p* > 0.05 following the transformation), as can also be seen in Table 1. This table shows the means and standard deviations of the transformed data (middle row), and the corresponding means after conversion back to the original scale (bottom row). All inferential statistics were conducted on the reciprocally transformed data.

**TABLE 1:** **Means and standard deviations (in parentheses) of responses in each condition of Experiment 1**.

	**300 ms**				**1000 ms**			
	**Easy**		**Hard**		**Easy**		**Hard**	
	**Cued**	**Uncued**	**Cued**	**Uncued**	**Cued**	**Uncued**	**Cued**	**Uncued**
Original data	384 (57)	400 (49)	540 (146)	527 (111)	371 (54)	373 (51)	514 (110)	500 (99)
Transformed data	0.002665 (0.00041)	0.002538 (0.00034)	0.001973 (0.00049)	0.001985 (0.00045)	0.002755 (0.00041)	0.002735 (0.00039)	0.002036 (0.00046)	0.002083 (0.00044)
Transformed data (original scale)	375	394	507	504	363	366	491	480
% correct	99.5	99.6	97.3	97.8	99.8	99.8	97.5	97.4

The units on the original scale are milliseconds. Units on the transformed scale are milliseconds^–1^. The table also shows percentage of correct responses in each condition.

The transformed data were subjected to an analysis of variance (ANOVA) with cue validity, secondary task and SOA as repeated measures factors. There was a significant main effect of secondary task *F*(1, 23) = 72.89, *p* < 0.001, ${\text{η}}_{p}^{2}$ = 0.76 reflected by overall slowing of reaction times under the hard secondary task condition (*M* = 495 ms) in comparison with the easy task (*M* = 374 ms). There was also a significant main effect of SOA, *F*(1, 23) = 18.86, *p* < 0.001, ${\text{η}}_{p}^{2}$ = 0.45 with faster reaction times to targets appearing 1000 ms after the onset of the gaze cue (*M* = 416 ms) than after 300 ms (*M* = 436 ms). The effect of cue validity did not reach significance, *F*(1, 23) = 2.06, *p* = 0.17, ${\text{η}}_{p}^{2}$ = 0.08, showing that, overall, participants responded no faster to cued targets (*M* = 424 ms) than uncued targets (*M* = 428 ms). However, the main effects were qualified by a significant interaction between task and cue validity, *F*(1, 23) = 6.85, *p* < 0.05, ${\text{η}}_{p}^{2}$ = 0.23, confirming that there was a modulation of the gaze cueing effect by the secondary task demands. Simple main effects analyses revealed that, under easy secondary task conditions, cued targets (*M* = 369 ms) were located faster than uncued targets (*M* = 379 ms), *F*(1, 46) = 8.69, *p* < 0.01, but that under hard secondary task conditions, performance for cued targets (*M* = 499 ms) was equivalent to that of uncued targets (*M* = 492 ms), *F*(1, 46) = 1.42, *p* = 0.24.

Finally, the ANOVA revealed a marginally significant interaction between cue validity and SOA, *F*(1, 23) = 3.79, *p* = 0.06, reflecting the observation that at the 300 ms SOA cued targets (*M* = 431 ms) were responded to faster than uncued targets (*M* = 442 ms), but at the 1000 ms SOA, the trend was in the opposite direction, with slightly faster location of uncued targets (*M* = 415 ms) than cued targets (*M* = 418 ms). No other interactions reached significance (*p*s > 0.13)^1^.

The percentages of correct responses are also shown in Table 1. It is clear from these data that participants were able to perform the target localization task very well indeed, making errors on just 1.4% of trials. Moreover there is no evidence of a trade off between speed and accuracy that would compromise interpretation of the RT data. As performance was essentially at ceiling level in all conditions, no further analyses were conducted on these data.

### Discussion

The overall pattern of the data indicated a cueing effect under easy dual task conditions, which disappeared when participants were engaged in an executively demanding secondary task. Participants were also slower and somewhat less accurate at target localization under hard relative to easy secondary task conditions, which suggests that generating random number sequences is indeed a more demanding task than reciting ordered sequences of digits. However, although participants’ accuracy was slightly lower under hard secondary task conditions, it was still very high indeed, suggesting that participants did not simply abandon the target localization task, or avert their gazes from the screen when performing the demanding secondary task. One possibility, however, is that participants may have maintained relatively high accuracy at target localization under difficult secondary task conditions by compromising their performance in generating random numbers. For example, they might have waivered from the requirement to generate numbers at the rate of one per second, or they may not have maintained an acceptable level of randomness. As we did not analyze these data we cannot address this possibility directly. The available data do suggest, however, that the RNG task had a detrimental effect on gazecued orienting. So, whether or not participants strayed from the maximum demands of the RNG task, it was still sufficient to disrupt gaze-cued orienting relative to performance in the easy secondary task condition.

The results of Experiment 1 imply that those mechanisms that are involved in the generation of random number sequences are also involved in the generation of an attention shift in response to a seen gaze. A key assumption underlying this interpretation of the data is that the difference in RTs for the localization of uncued versus cued targets is caused by the allocation of visual attention in response to the gaze cue. However, an alternative interpretation is that the RT difference between uncued and cued conditions could actually reflect a difference in the degree of stimulus-response compatibility between these cases. The argument is as follows. First, there is evidence that gazes and other social cues automatically trigger the generation of spatial codes (Langton et al., 1996; Langton, 2000; Langton and Bruce, 2000). It is reasonable to assume, therefore, that the gaze cues in the present experiment also trigger the generation of such codes. On cued trials, the gazes would result in the generation of spatial codes which are the same as those required for the key press responses (e.g., gaze right, target right); under uncued conditions, these codes would be different (e.g., gaze right, target left). The RT difference between uncued and cued conditions could therefore be the result of difficulties in response selection, for example, rather than any shifting of visuo-spatial attention. The interaction effect that we have observed in Experiment 1 might therefore reflect the influence of RNG on response selection processes, rather than on gaze-cued orienting of attention. This problem was addressed in Experiment 2.

## Experiment 2

In order to eliminate a response selection account for the cueing effect observed in Experiment 1, in Experiment 2 we used a target identification, rather than a target localization task. Additionally, we also included a condition that ought to be immune from a demanding secondary task—one where the identity of a target is assessed as a function of whether or not its location has been indicated by a peripheral luminance change.

### Materials and Methods

#### Participants

Undergraduates from the University of Stirling (*N* = 32, 14 female, 18 male) were recruited for this experiment. They received course credit for participation. The mean age was 21.59 years (range: 18–44 years).

#### Materials and Apparatus

These were identical to those used in Experiment 1 in all but the following respects. The target stimuli for both the gaze cueing and peripheral cueing tasks comprised the letters T and F in 18 point Arial font. In the peripheral cueing task, two grey boxes appeared centered 4.1° to the left and right of the central fixation cross. The lines of these boxes were 1 pixel thick and the boxes measured 1.6° in height and 1.4° in width. The spatial cue in this condition was rendered by replacing one of the grey placeholder boxes with an identically sized white box, the lines of which were six pixels thick.

#### Design

The experiment had a 2 × 2 × 2 design with cue type (gaze cue, peripheral cue) as a between-subjects independent variable and cue validity (cued, uncued), and task type (hard, easy) as within-subjects variables. SOA was not manipulated in this experiment and was instead fixed at 300 ms for both cue types. This SOA produced the largest magnitude of gaze-cueing in Experiment 1, and is also short enough to elicit a cueing effect from peripheral onsets (Müller and Rabbitt, 1989).

#### Procedure

The easy and hard secondary tasks were identical to those used in Experiment 1. The procedure for gaze-cueing trials was identical to that of Experiment 1, save for the facts that the SOA was fixed at 300 ms for all trials, targets comprised the letters T and F, and participants were asked to identify the target letter on each trial by pressing the topmost button on the response box for the letter T and the bottom button for the letter F.

Trials in the peripheral cue condition began with a 2000 ms presentation of the display comprising the fixation cross and placeholders. One of the placeholder boxes was then replaced by the white cue box. The target letter (T or F) appeared centered in either the cued box, or the uncued box 300 ms after the onset of the cue, and remained on the screen until the participant had responded.

Participants completed 64 trials under each secondary task condition, divided into two blocks of 32 trials. A block of 16 practice trials preceded each pair of experimental blocks. The order in which participants completed each pair of easy and hard secondary task blocks was counterbalanced across participants, and participants were randomly allocated to either the gazecueing or peripheral cueing task, with the constraint that an equal number took part in each task.

### Results

Participants made errors on 4% of all gaze-cueing trials in Experiment 2 and these responses were removed from subsequent analyses of the RT data. Median RTs were then computed as in Experiment 1, and the interparticipant means and standard deviations of these data are presented in Table 2. Once again, because of the heterogeneity of variance evident in the data (Hartley’s *F*_max_ = 18.84, *p* < 0.01), RTs were subjected to a reciprocal transform, which was found to stabilize the variances across experimental conditions (Hartley’s *F*_max_ = 1.93, *p* > 0.05). The means and standard deviations of these transformed data are also presented in Table 2, along with the corresponding untransformed means. As in Experiment 1, all inferential statistics were conducted on the reciprocally transformed data.

**TABLE 2:** **Means and standard deviations (in parentheses) of responses in each condition of Experiment 2**.

	**Gaze cues**				**Peripheral cues**			
	**Easy**		**Hard**		**Easy**		**Hard**	
	**Cued**	**Uncued**	**Cued**	**Uncued**	**Cued**	**Uncued**	**Cued**	**Uncued**
Original data	467 (74)	489 (78)	619 (181)	634 (224)	438 (52)	533 (63)	566 (131)	648 (159)
Transformed data	0.002182 (0.00027)	0.002086 (0.00027)	0.001737 (0.00045)	0.001718 (0.00045)	0.002311 (0.00025)	0.001901 (0.00024)	0.001847 (0.00038)	0.001631 (0.00045)
Transformed data (original scale)	458	479	576	582	433	526	541	613
% correct	96.1	96.4	94.9	95.6	97.9	95.1	96.3	94.2

The units on the original scale are milliseconds. Units on the transformed scale are milliseconds^–1^. The table also shows percentage of correct responses in each condition.

An ANOVA was conducted on the reciprocally transformed RT data, with secondary task (easy vs. hard), and cue validity (cued vs. uncued) as repeated measures factors, and cue-type (gaze vs. peripheral) as a between-subjects factor. This analysis yielded a main effect of secondary task, *F*(1, 30) = 62.03, *p* < 0.001, ${\text{η}}_{p}^{2}$ = 0.67, with faster identification of targets under easy secondary task conditions (*M* = 472 ms) than hard secondary task conditions (*M* = 577 ms). There was also a main effect of cue validity, *F*(1, 30) = 62.17, *p* < 0.001, ${\text{η}}_{p}^{2}$ = 0.68, reflecting faster performance for cued targets (*M* = 495 ms) than uncued targets (*M* = 545 ms). However, these main effects were qualified by interactions between secondary task and cue validity, *F* (1, 30) = 24.66, *p* < 0.001, ${\text{η}}_{p}^{2}$ = 0.45, cue validity and cue-type, *F* (1, 30) = 29.74, *p* < 0.001, ${\text{η}}_{p}^{2}$ = 0.50, and by all three factors, *F* (1, 30) = 4.62, *p* < 0.05, ${\text{η}}_{p}^{2}$ = 0.13.

In order to explore the significant 3-way interaction, separate repeated measures ANOVAs were conducted on the RT data from the group who performed the gaze-cueing primary task and those who performed the peripheral cueing task, each with cue validity and secondary task as factors.

#### Gaze-cueing Task

For the group performing the gaze cueing trials, the ANOVA yielded significant main effects of secondary task, *F*(1, 15) = 26.17, *p* < 0.01, ${\text{η}}_{p}^{2}$ = 0.64, and cue validity, *F*(1, 15) = 6.74, *p* < 0.05, ${\text{η}}_{p}^{2}$ = 0.31, and a significant interaction between these factors, *F*(1, 15) = 4.54, *p* = 0.05, ${\text{η}}_{p}^{2}$ = 0.23. Simple main effects analyses indicated that under easy secondary task conditions, participants were faster to identify cued targets (*M* = 458 ms) than uncued targets (*M* = 479 ms), *F*(1, 30) = 11.28, *p* < 0.01; however, there was no such cueing effect under hard secondary task conditions (cued targets: *M* = 576 ms; uncued targets: *M* = 582 ms), *F*(1, 30) = 0.44, *p* = 0.51.

#### Peripheral Cueing Task

The equivalent analysis conducted on the data from participants who performed the peripheral cueing trials yielded main effects of secondary task, *F*(1, 15) = 40.39, *p* < 0.001, ${\text{η}}_{p}^{2}$ = 0.73, and cue validity, *F*(1, 15) = 56.98, *p* < 0.001, ${\text{η}}_{p}^{2}$ = 0.79, and a significant interaction between these factors, *F*(1, 15) = 22.48, *p* < 0.001, ${\text{η}}_{p}^{2}$ = 0.60. Subsequent simple main effects analyses confirmed that the effects of cue validity were reliable under both easy secondary task conditions (cued targets: *M* = 433 ms; uncued targets: *M* = 526 ms), *F*(1, 30) = 78.60, *p* < 0.001, and hard secondary task conditions (cued targets: *M* = 541 ms; uncued targets: *M* = 613 ms), *F*(1, 30) = 21.93, *p* < 0.001 with the interaction presumably arising because the magnitude of the cueing effect was larger under the former (93 ms) than the latter (72 ms)^2^.

The percentage of correct responses are also shown in Table 2. Participants were clearly performing at a high level of accuracy and there is no evidence of a trade off between speed and accuracy that would compromise interpretation of the RT data. No further analyses were conducted on these data.

### Discussion

In Experiment 2 all participants performed a target identification task instead of the target localization task used in Experiment 1. For half of the participants, spatial cues were provided by a gaze shift, as in Experiment 1, whereas peripheral luminance transients formed the cues for the remaining participants. Once again, participants carried out the gaze-cueing task, or peripheral orienting task while simultaneously performing an easy secondary task in some blocks of trials, and a hard secondary task RNG in others. Results indicated significant cueing effects under the easy secondary task conditions for both types of cue; however, the gaze cueing effect, but not the peripheral cueing effect, was eliminated when participants simultaneously performed the executively demanding RNG task. This finding supports the conclusion from Experiment 1 that gaze-cued orienting of attention and RNG involve at least some of the same cognitive mechanisms.

One curious aspect of the data is the observation that the peripheral cueing effect was actually reduced, though not eliminated, under hard secondary task conditions. Peripheral luminance changes are thought to capture attention in a purely stimulus-driven fashion (e.g., Jonides and Yantis, 1988; Yantis and Jonides, 1990; Franconeri et al., 2005), so why should the cueing effect have been influenced at all by an executively demanding secondary task? One possibility is that under the easy secondary task conditions, the procedure allowed peripheral cues to trigger both an exogenous and an endogenous orienting of attention. Studies investigating the time courses of the two types of orienting suggest that each have distinct but overlapping time courses: orienting based on peripheral cues occurs rapidly and is strongest between 100 and 300 ms after cue onset, with a peak at around 150 ms; endogenous orienting is rather slower and reaches its peak at around 300 ms (e.g., Müller and Rabbitt, 1989; Cheal and Lyon, 1991). Thus, at the SOA of 300 ms used in Experiment 2, we might expect both kinds of attention to be deployed toward the target location, producing additive effects on RT under easy secondary task conditions. If RNG disrupts only endogenous orienting, this will still leave some facilitation caused by the rapid exogenous orienting of attention under the more difficult secondary task, as was observed.

A similar argument might be made for gaze-cued orienting. At an SOA of 300 ms the advantage for target identification at cued versus uncued locations could involve both an exogenous and an endogenous deployment of attention, with RNG disrupting only the latter. However, as we have observed, there is no residual cueing effect under difficult dual task conditions that could be attributed to exogenous factors. Therefore, the gaze-cueing effect observed under easy secondary task conditions is likely to be driven by some of the same endogenous mechanisms that are involved in RNG.

## General Discussion

The two experiments reported here investigated the extent to which gaze-cued orienting of attention is under top-down control. In each experiment, we assessed RT to targets whose location was cued by a gaze shift, relative to targets that appeared in a location opposite to that indicated by the direction of gaze. In order to assess the involvement of voluntary control in gaze cueing, performance was assessed while participants simultaneously completed an easy secondary task, and compared with performance while executing a demanding secondary task. With both a target localization (Experiment 1) and a target identification (Experiment 2) decision, a gaze cueing effect was observed when participants were simultaneously executing the undemanding secondary task—repeatedly reciting the digits 1–9 in sequence; however, gaze cueing was disrupted when participants were simultaneously generating random numbers. RNG is argued to place high demands on WM resources (e.g., Vandierendonck et al., 2004; Towse and Cheshire, 2007). The conclusion is therefore that these same resources are involved in the orienting of attention made on the basis of an observed shift in someone’s gaze. In other words, gaze-cued attention is not a strongly automatic process and is instead under a degree of top-down control.

The results obtained in these experiments contradict those of Law et al. (2010) and Hayward and Ristic (2013) who found that gaze-cued orienting was resistant to a secondary task load. However, as argued above, it may be that the secondary tasks used in these studies could be temporarily suspended while participants performed the gaze-cueing trials. Our data show that a WM task that runs fully in parallel with gaze cueing trials (i.e., it is not suspended at any point during the gaze cueing trials) does, indeed, disrupt the gaze cueing effect.

Should we therefore understand gaze-cued orienting to be simply another manifestation of volitional, endogenous orienting of attention—in other words, the deliberate allocation of attentional resources in response to current goals? The answer seems to be no. While our data suggest that gaze-cued orienting shares resources with whatever control processes are used in RNG, plenty of other data point to it being much more like a stimulus-driven effect—the allocation of resources based on factors external to the observer; for example, it is observed even when gazes are known to be uninformative or even counter-informative of the likely location of an upcoming target (see Frischen et al., 2007). Indeed, at least two studies have shown that attention can be deployed volitionally toward a location opposite to that indicated by a gaze cue, at the same time as being deployed in the direction indicated by the direction of gaze (Friesen et al., 2004; Hayward and Ristic, 2013). These data suggest that gaze-cued attention and volitional orienting are independent of one another.

So, gaze-cued attention should not be thought of as another example of a purely volitional process (i.e., endogenous orienting), but then neither can it be described as a stimulus-driven reflex (i.e., exogenous orienting). Stimulus-driven processes occur whenever their triggering stimuli are present, and are resistant to concurrent load manipulations. The data reported here suggest that, in contrast, gaze-cued orienting *is* influenced by a concurrent WM load. Gaze-cued attention therefore clearly bears a resemblance to exogenous orienting as well as to endogenous forms of orienting. The difficulty, then, is generating a theory that can account for these seemingly contradictory observations.

Ristic and Kingstone’s (2012) solution to the dilemma is that gazes, arrows and words with spatial meaning engage a unique mechanism called *automated symbolic orienting*, which occurs without intention, and arises as a result of the overlearning of associations between cues and target events. Our proposal is different in that it acknowledges a specific role for a top down mode of control in gaze-cued orienting. We suggest that orienting to gazes occurs as a result of an internally generated goal that is maintained by top-down signals from the WM. This goal might be characterized by the rule “look where others look” and may arise through, for example, learning about contingencies between gazes and rewarding target events, a suggestion originally made by Langton and Bruce (1999) and Driver et al. (1999) to explain their observations of gaze-cued orienting.

The key idea is that “look where others look” is a goal state that is almost permanently maintained by top-down signals that activate mechanisms involved in detecting and responding to the appropriate environmental trigger (a gaze shift, for example). This top-down activation is what gives gaze-cued orienting its resemblance to endogenous attentional control. However, because of this top-down activation, any stimulus that meets the relevant criteria (e.g., moving eyes or eye-like stimuli) will trigger the associated behavior (an attention shift). This attention shift occurs as long as the default goal state remains undisrupted by other, highly demanding attentional goals that engage WM concomitantly.

Notably, the gaze-cued orienting effect will persist even in the face of concurrent task demands, as long as the concurrent task does not recruit the same top-down mechanisms that are involved in maintaining the “look where others look” goal state. Repeatedly counting from 1 to 9 is a well practiced routine, which does not require the generation and maintenance of complex stimulus-response mappings, establishment of novel module-to-module couplings, iterative monitoring and modification of performance and so on. Maintaining a digit load in WM may be similarly untaxing, as it relies on a dedicated component of WM (e.g., the phonological loop in the WM model, see Baddeley, 2000) and it is unclear whether it is performed in parallel with the gaze cueing trials. RNG, on the other hand, requires much more in the way of controlled processing. One must first generate a strategy in order to produce the desired output; representations of the possible response alternatives must be activated and maintained in WM so that they are available for selection; the output must be monitored in relation to some internally generated concept of randomness; and it is likely that inhibitory processes act to suppress the generation of overlearned sequences (Towse and Cheshire, 2007). These might be thought of as a number of sub-goals that must be generated and maintained in order to satisfy the main task goal of generating the random sequence. We suggest that it is this requirement that swamps the ability to maintain the goal of looking where others look (cf. Duncan et al., 1996).

This theory suggests that it is the number of simultaneously active sub-goals required of RNG that disrupts the orienting of attention to seen gazes; however, it is of course possible that the source of interference is one or more of the component processes themselves. Further research will be required to explore this possibility. The theory also presents a solution to another puzzle: if gaze-cued orienting were truly a stimulus-driven process, it ought to occur every time a gaze shift is viewed, and would likely be accompanied by an overt shift in gaze as covert and overt orienting usually, but not inevitably, occur in tandem (see Findlay and Gilchrist, 2003); yet automatic *overt* attention shifts in response to others’ gazes patently do not occur outside the confines of the laboratory. How is it that averted gazes that when seen in the laboratory readily trigger covert attention shifts do not seem to trigger overt shifts in more naturalistic situations? The answer may be that gazes seen in natural situations simply do not tend to trigger covert shifts of attention due to high cognitive demand imposed by social situations in which these gazes occur. Indeed, covert gaze-cueing might be observed in the laboratory where participants’ concurrently active goals are reduced to the generation and maintenance of relatively straightforward stimulus-response mappings (e.g., press the top button for a letter T, the bottom button for a letter F); however, the effect may vanish in many normal interactions in which participants tend to have multiple, continuously changing concurrent goals. Pertinently, in their recent study, Gregory et al. (2015) showed that when viewing a “live” scene with socially engaged actors, overt attention to gazes and heads is reduced (cf. Freeth et al., 2013). The authors explain their findings in terms of a cognitive load that is required for processing bodies, and making higher cognitive judgements about the presented social scene. This load disrupts “reflexive” shifts of attention present in viewing gazes passively such as in a laboratory environment. It is possible that the secondary task used in our studies produced similarly high cognitive demands for the WM system to stop prioritizing gazes.

An alternative explanation for our data is that rather than imposing high general cognitive demands, RNG exerts its effects on gaze cued orienting specifically through disrupting the spatial processing involved in extracting gaze direction from the eyes and executing an attention shift in the computed direction. In support of this suggestion, it is well known that the mental representations of numbers are associated with spatial codes (e.g., Zorzi et al., 2002), with low numbers associated with the left side of space and high numbers with the right side of space (Dehaene et al., 1993). Pertinently, there is also a large body of research showing that parietal cortex is involved in numerical representations in humans and primates (see Nieder, 2004, for a review) and that gaze cued attentional orienting is also mediated by lateral parietal regions of the brain (see Carlin and Calder, 2013, for a review).

The proposal is, then, that the same spatial processing resources may be involved in gaze-cued orienting and RNG. This is an intriguing suggestion as it could account for why RNG disrupts gaze cued orienting, whereas other high load tasks do not. It is not immediately obvious, however, why the generation of numbers in an ordered sequence in our easy secondary tasks would not also involve the same spatial resources as does generating the same digits in a random order. Indeed, one might argue that spatial coding is actually stronger in the case of ordered number generation as one can readily imagine the ordered sequence in a number line from left to right. On this view it seems likely that any spatial coding induced by the generation of numbers is controlled across the secondary tasks used in our experiments. In support of a spatial account, it could be argued that RNG draws more heavily on spatial resources than does ordered number generation, for the latter simply involves reading off a stereotyped verbal sequence, which might not involve the activation of individual spatial representations to the same extent as RNG. Indeed, numbers are likely associated with different kinds of representations—verbal as well as visuo-spatial—with different representations deployed according to the nature of the number-involving task (e.g., van Dijck et al., 2009). Given this, it is of course possible that neither secondary task involves the activation of spatial codes; both random and ordered number generation may involve verbal rather than spatial coding of numbers. According to this account, neither task would impact upon gaze-cued orienting through drawing upon a limited spatial resource.

Our data do not allow us to tease apart these possibilities directly, although the fact that the spatial location of the target interacted with neither secondary task nor cue validity hints that spatial coding may not be a crucial factor^1,2^. Nevertheless, the suggestion that RNG exerts its effects on gazed-cued orienting through a spatial mechanism is clearly one that warrants further research.

In summary, in two experiments, we assessed the effects of a concurrent WM demand on social orienting. Our main finding was that social attention was disrupted by the RNG task. Data from this study stands in contrast to previous laboratory-based findings in suggesting that attention cued by gazes is, indeed, dependent on top-down control.

### Conflict of Interest Statement

The authors declare that the research was conducted in the absence of any commercial or financial relationships that could be construed as a potential conflict of interest.

## Footnotes

1 In order to examine whether the source of the interference effect of RNG on gaze cued orienting might be an incompatibility between the spatial code generated by the appearance of the target and one that might be associated with the generation of random numbers (e.g., producing number sequences from left to right in visual imagery), we also performed an ANOVA with target location (left vs. right) as an additional repeated measures factor. However, target location was found to interact with neither of the other two factors, and nor did the predicted interaction between target location, secondary task and cue validity reach statistical significance (*p* = 0.84).

2 As with Experiment 1, we also performed an ANOVA including target location (left vs. right) as an additional repeated measures factor, but again this analysis failed to yield any significant effects involving this factor (*p*s > 0.14).
